# Heterologous Expression of Arabidopsis *AtARA6* in Soybean Enhances Salt Tolerance

**DOI:** 10.3389/fgene.2022.849357

**Published:** 2022-05-12

**Authors:** Zhipeng Hong, Yang Li, Yang Zhao, Mingyu Yang, Xiaoming Zhang, Yuhan Teng, Linjie Jing, Danxun Kong, Tongxin Liu, Shuanglin Li, Fanli Meng, Qi Wang, Ling Zhang

**Affiliations:** ^1^ Key Laboratory of Soybean Biology in Chinese Ministry of Education, Northeast Agricultural University, Harbin, China; ^2^ Institute of Crop Cultivation and Tillage, Heilongjiang Academy of Agricultural Sciences, Harbin, China; ^3^ Agro-Biotechnology Research Institute, Jilin Academy of Agricultural Sciences, Changchun, China

**Keywords:** soybean, *AtARA6*, salt tolerance, RAB GTPase, RAB5, SNARE pathway

## Abstract

Salt damage is an important abiotic stress affecting the agronomic traits of soybean. Soybeans rapidly sense and transmit adverse signals when salt-damaged, inducing a set of response mechanisms to resist salt stress. *AtARA6* encodes a small GTPase, which plays an important role in Arabidopsis vesicle transport and salt tolerance. In this study, we transformed the Arabidopsis gene *AtARA6* into the cultivated soybean Shen Nong 9 (SN9). To investigate the salt tolerance pathways affected by *AtARA6* in soybean, we performed transcriptome sequencing using transgenic soybean and wild-type (SN9) under salt treatment and water treatment. Our results suggest that *AtARA6* is involved in the regulation of soybean SNARE complexes in the vesicle transport pathway, which may directly strengthen salt tolerance. In addition, we comprehensively analyzed the RNA-seq data of transgenic soybean and SN9 under different treatments and obtained 935 DEGs. GO analysis showed that these DEGs were significantly enriched in transcription factor activity, sequence-specific DNA binding, and the inositol catabolic process. Three salt-responsive negative regulator transcription factors, namely *MYC2*, *WRKY6*, and *WRKY86*, were found to be significantly downregulated after salt treatment in transgenic soybeans. Moreover, four genes encoding inositol oxygenase were significantly enriched in the inositol catabolic process pathway, which could improve the salt tolerance of transgenic soybeans by reducing their reactive oxygen species content. These are unique salt tolerance effects produced by transgenic soybeans. Our results provide basic insights into the function of *AtARA6* in soybeans and its role in abiotic stress processes in plants.

## Introduction

Soybean (*Glycine max* [L.] Merr.) is an important oilseed crop that provides high-quality nutrients and is rich in proteins, unsaturated fatty acids, and many bioactive substances, such as isoflavones. Soybean oil is not only used as food but also as a fuel (Qi and Lee, 2014). Due to the diversity of uses, the demand for soybeans is continuously increasing. However, soybean yields are chronically compromised by abiotic stresses, such as drought and salt stress (Phang et al., 2008; Manavalan et al., 2009). Soil salinity affects 20% of the world’s arable land and severely affects soybean yield and quality (Hamwieh and Xu, 2008; Wang et al., 2010). Currently, more than 100 countries worldwide are facing serious soil salinization (Tester and Davenport, 2003; Munns and Tester, 2008). To address this problem, the search for salt-tolerant genes and the breeding of salt-tolerant soybean varieties have become a research priority for breeders.

A variety of techniques have been used to breed salt-tolerant soybean. While traditional breeding methods have limitations due to long cycle times, transgenic techniques can quickly yield soybean germplasm with good agronomic traits and genetic stability, through overexpression or downregulation of target genes. With the advent of advanced molecular biology techniques, salt tolerance genes and salt tolerance mechanisms have been widely studied. Many genes can improve salt tolerance in soybeans by coordinating ion transport. For example, overexpression of the Arabidopsis *AtNHX* (Na^+^, K^+^/H^+^ exchangers) antiporter gene, *AtNHX5*, enhances transgenic soybean salt resistance by increasing the concentration of Na^+^ in leaves and roots (Wu et al., 2016).

Drought and salt damage are the most common stressors that have a significant impact on plant growth and development, causing significant losses in crop yield (Beeson et al., 2007; Tester and Langridge, 2010; Agarwal et al., 2013). Plants have multiple ways to deal with abiotic stresses, such as vesicular transport. Substantial evidence suggests that vesicular trafficking ensures the efficient transport of stress-related ions to vacuoles. For example, the involvement of *SchRabGDI1* in endocytic transport and tolerance to salt stress in *Solanum chilense* has been reported (Martin-Davison et al., 2017).

In eukaryotic organisms, membrane transport is a conserved cellular process that moves materials between organelles. This process occurs in three steps: vesicle budding, shuttling between organelles, and fusion. Small extracellular molecules are first screened by clathrin, then the donor cell membrane sags to form vesicles through endocytosis. The vesicles first bud from the donor membrane and then move along the cytoskeleton to the target membrane, where they undergo docking and tethering processes, among others. Finally, the fusion of vesicles and target membranes mediated by SNARE proteins complete material transport. As a house-keeping process, it is tightly regulated. Membrane transport mediated by vesicles/tubules is an important component of substance transport in plants. RAB-GTPase, a small GTPase with a molecular size of 20–25 kDa, is involved in vesicular transport and functions as a molecular switch in the cycling between GDP and GTP (Bucci and Chiariello, 2006; Schwartz et al., 2007). RAB5 belongs to the RAB family. Arabidopsis has three RAB5 homolog genes, *AtARA7* (RABF2b), *AtRHA1* (RABF2a), and *AtARA6* (RABF1) (Ueda et al., 2001). Both *AtARA7* and *AtRHA1* are localized in the perivacuolar compartment (PVC), while *AtARA6* is localized at the plasma membrane, in the Golgi network (TGN), and in multivesicular endosomes (MVEs), suggesting that it may be involved in intracellular endosomal transport (Hoepflinger et al., 2013). In the TGN, *AtARA6* affects SNARE complexes (soluble N-ethylmaleimide-sensitive factor attachment protein receptor), which are crucial for the fusion step, by regulating the R-SNARE/VAMP727 complex on the endosome and the Q-SNARE/SYP121 complex on the plasma membrane (Ebine et al., 2011).

The importance of the SNARE pathway has been investigated under salt stress conditions (Singh et al., 2016). In Arabidopsis, *the AtARA6* knockout line has a moderately salt-sensitive phenotype, while *the AtARA6*-overexpression line has enhanced salt tolerance (Ebine et al., 2011). *SYP121* functions in drought and ABA signaling pathways in tomato (Leyman et al., 1999). Disruption of *SYP121* decreases the water permeability of the cell membrane (Besserer et al., 2012). In Arabidopsis, *the SYP121* mutant, *AtSYP121*, shows suppressed stomatal opening, indicating that the gene may be involved in the drought response (Eisenach et al., 2012). Recently, *VAMP727* was found to physically interact with *BRI1*, a brassinosteroid receptor (Zhang et al., 2019b), thus responding to broad stress stimuli. Another RAB family member, Rab7, has been shown to negatively affect salt tolerance in Arabidopsis (Mazel et al., 2004).

*AtARA6* is different from other RAB5 members because of its unique N-terminal myristoylation and palmitoylation, but it has a common activator, *VPS9a* (Grosshans et al., 2006). To date, *AtARA6* homologues have been found in all sequenced plants, except for single-celled green algae, and have not been found in animals (Ebine et al., 2012). *AtARA*6 exhibits a variety of biological functions, including endosomal transport, signal transduction, stress response, and growth regulation. In this study, we heterologously expressed the *AtARA6* gene in soybean and found that salt tolerance was enhanced (Supplementary Figure S1). We performed RNA-seq to uncover potential pathways affected by the *AtARA6* transgene. Our study provides insights into the function of *AtARA6* and lays a foundation for breeding new germplasms of transgenic salt-tolerant soybean.

## Materials and Methods

### Plant Material and Growing Environment

Col-0 wild-type Arabidopsis was incubated in a normal growth chamber with a light/dark cycle of 16 h/8 h at 22°C/16°C. Transgenic soybean and SN9 were cultured in a greenhouse environment with a light/dark cycle of 16 h/8 h at 28°C/25°C.

### Construction of Expression Vector and Soybean Genetic Transformation

Total RNA was extracted from 3 week-old wild-type Arabidopsis leaves and was reverse transcribed into cDNA using the PrimeScript RT kit (Takara, Shiga, Japan). The 609 bp CDS of Arabidopsis *AtARA6* (AT3G54840) was amplified with primers (5′-GAA​GAG​AAG​AAG​CAC​ATC​CCA​T-3′ 5′- ATG​GGA​TGT​GCT​TCT​TCT​CTT​C-3′) designed using Primer 5.0. The amplification products were recovered using a DNA Gel Extraction Kit (Tiangen Biotechnology Co. Ltd., Beijing, China) and sent for sequencing. The *AtARA6* CDS was cloned into the pTF101 vector *via* Spe1 and Sac1 sites. The plasmid was transformed into *Agrobacterium tumefaciens* strain EHA101. Cultivated soybean SN9 was infected with the strain by the cotyledon nodulation method. Regenerated plants were obtained after exosome infiltration, clump shoot induction, clump shoot screening, and rooting culture (Paz et al., 2006; Zhang et al., 2019a). The *bar* gene driven by the CaMV35S promoter in the PTF101 vector was used for the positive progeny screen.

### Identification of Transgenic Positive Progeny

The transgenic progeny was first tested using LibertyLink test strips, according to the manufacturer’s instructions (EnviroLogix Inc., Portland, ME, United States). Genomic DNA from test strip-positive soybean leaves was extracted using a DNA extraction kit (Kangwei Century Biotechnology Co., Ltd., Beijing, China), and primers were designed according to the CDS sequences of *AtARA6* and *Bar* genes. The soybean actin gene was used as a control (Supplementary Table S1).

### Salt Stress Treatment

The transgenic soybeans and recipient soybean seeds were sowed in vermiculite and watered with 0, 100 mM, or 200 mM NaCl solutions (Wang et al., 2018). Root length was measured after 1 week. For the salt stress treatment at emergence stages, the NaCl concentrations chosen followed the method of Li et al. (2017), with a slight modification: we first sowed the seeds in vermiculite and treated them with water for 10 days until the main leaves fully unfolded, then they were treated with 200 mM NaCl. The salt solution was changed every 3 days. Soybean phenotypes were recorded at weeks 1, 2, and 3 after treatment.

### Determination of Salt Tolerance Parameters

We measured representative physiological indicators of salt tolerance in transgenic lines and SN9, including superoxide dismutase (SOD), peroxidase (POD), and catalase (CAT) activities after 3 weeks of salt treatment. SOD activity was determined based on the photochemical reduction capacity of p-Nitro-Blue tetrazolium chloride, and the absorbance value was read at 560 nm (Beauchamp and Fridovich, 1971). POD activity was determined based on the amount of reaction per unit time using guaiacol as a substrate (Reuveni, 1992). During the disappearance of H_2_O_2_, the activity of CAT is detected by monitoring the change in absorbance at 240 nm (Yang et al., 2008). Malondialdehyde (MDA) reflects the level of peroxidation of plant cell membrane lipids, and we measured MDA contents to assess the degree of damage to transgenic lines and WT plants (Landi, 2017). Chlorophyll was extracted in a mixture of acetone and ethanol, and the absorbance was measured at 645, 652, and 663 nm (Arnon, 1949). The determination of proline was based on the method described by Irigoyen et al. (1992).

### RNA Extraction, Library Construction, and RNA Sequencing

The transgenic positive line2 and WT, after salt stress treatment for 5 days, were named S1 and S2, respectively. The transgenic positive line2 and WT, after water treatment for 5 days, were used as controls, and named H1 and H2, respectively. Total leaf RNA was extracted using the OminiPlant RNA Kit (Dnase I) (Kangwei Century Biotechnology Co., Ltd., Beijing, China), according to the manufacturer’s instructions. Transcriptome sequencing was performed at Biomarker Biotechnology (Beijing, China). The mRNA was enriched with magnetic beads with Oligo (dT), and cDNA was synthesized using random primers. The purified double-stranded cDNA was end-repaired, A-tail was added, sequencing connectors were ligated, and the cDNA library was obtained by PCR. The RNA-seq library was constructed using Illumina novaseq 6000.

### qRT-PCR Validation of Gene Expression

Leaves collected 5 days after salt treatment were used for qPCR validation. Total plant RNA was extracted using a Plant RNA Extraction Kit (Kangwei Century Biotechnology Co., Ltd., Beijing, China), and gDNA Remover Mix was used to remove genomic DNA contamination. An ABScript II RT Mix (ABClonal technology, Wuhan, China) was used for the reverse transcription reaction, according to the manufacturer’s instructions. To investigate the expression of *AtARA6* at different treatment times and the expression of genes with which *AtARA6* may have a reciprocal relationship, gene primers and GmActin (NM_001289231) primers were designed using the NCBI online website (Supplementary Table S2). The cDNA was amplified using SYBR Green Fast qRT-PCR Mix (ABClonal Technology Co. Ltd., Wuhan, China) using the chimeric fluorescence method. Each treatment had three replicates. Two internal reference primers of GmActin were used to ensure the accuracy of qPCR. qPCR experiments were performed using the LightCycler 480 II machine. Relative expression of GmActin was calculated using the 2^−ΔΔCT^ method.

### Data Quality Control and Read Mapping

Raw data generated by sequencing were processed using FastQC and Trim_galore software (the quality control threshold was 25, reads with a length lower than 50 were discarded, the allowable error rate was 0.1, and the minimum number of bases overlapping with the adapter sequence was 3) to obtain high-quality data for mapping (reads with a ratio of N greater than 10% and reads (Q ≤ 10%) whose bases accounted for more than 50% of the entire reads were removed). The Williams 82 reference genome was constructed using HISAT2 software (version 2.1.0) (Parameter; hisat2-build -p 4 W82_ref. fa -snp W82_ref.snp -ss W82_ref. ss -exon W82_ref. exon W82_hisat2_index) (Kim et al., 2015), and unique reads were compared. Reads on pairs were assembled and quantified by DESeq2 (the test method was “Wald significance tests” and the fit type was “parametric”) using the StringTie comparison (the TPM value for the minimum allowed input transcript was 0.1 and the minimum isoform fraction was 0.1) (Love et al., 2014; Pertea et al., 2015). StringTie used FPKM (transcript per million fragments mapped) as a measure of transcript or gene expression level. Upregulated and downregulated genes between transgenic soybean and SN9 under salt treatments were determined using the edgeR package in R (Robinson et al., 2010), and all analyzed False Discovery Rate (FDR) thresholds were less than 0.05 (Fold Change ≥ 2). All raw data have been uploaded to the SRA database in NCBI (ID: PRJNA789350).

### Gene Ontology and KEGG Pathway Enrichment

Differentially expressed genes (DEGs) generated between each treatment were analyzed for gene ontology (GO) gene function clustering and enrichment via online websites (http://www.geneontology.org and http://bioinfo.cau.edu.cn/agriGO) with an FDR threshold set at 0.05 (Huang da et al., 2009; Du et al., 2010). Gene ontology was based on the National Center for Biotechnology Information (NCBI) non-redundant protein sequences (Nr), Swiss-Prot, protein family, and clusters of orthologous groups of proteins (KOG/COG) database. KEGG [Kyoto Encyclopedia of Genes and Genomes (KEGG) and Ortholog database (KO)] pathway enrichment analysis of DEGs was performed using the (http://www.genome.jp/kegg) website (Kanehisa et al., 2021), and we screened for pathways with *p* values less than 0.05.

All data were analyzed using two-way Analysis of Variance to evaluate significant differences at *p < 0.05, **p < 0.01, and ***p < 0.001. All statistical analyses were carried out IBM SPSS Statistics 22 (IBM Corp., Armonk, NY, United States). All DEGs and pathways used for GO analysis and KEGG analysis can be found in Supplementary Table S3 and Supplementary File S1.

## Results

### Soybean Transformation and Positive Line Identification

**FIGURE 1:**
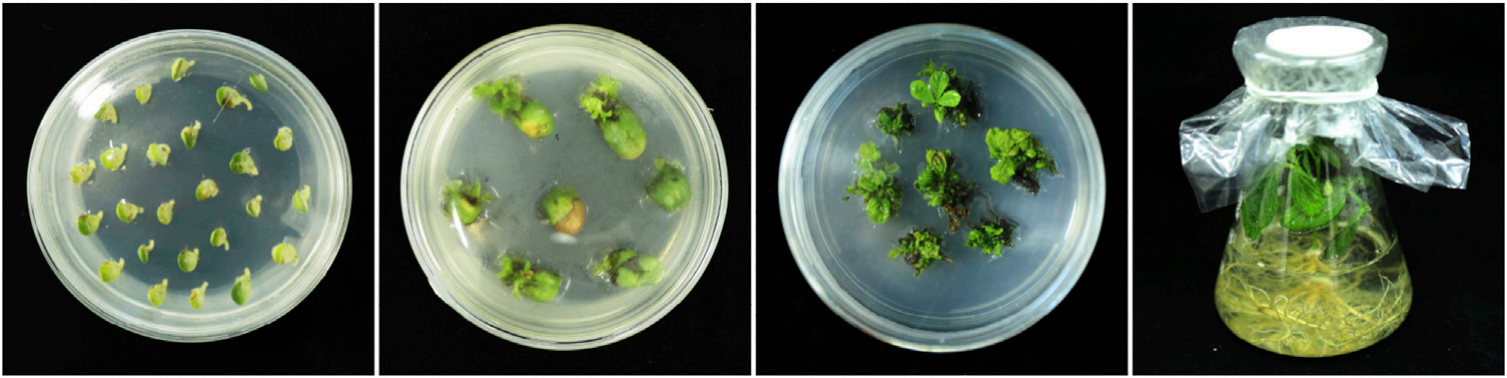
Genetic transformation of *AtARA6*. Agrobacterium tumefaciens EHA101-mediated genetic transformation of soybean, including exosome infiltration, clump shoot induction, clump shoot screening, and rooting culture.

**FIGURE 2:**
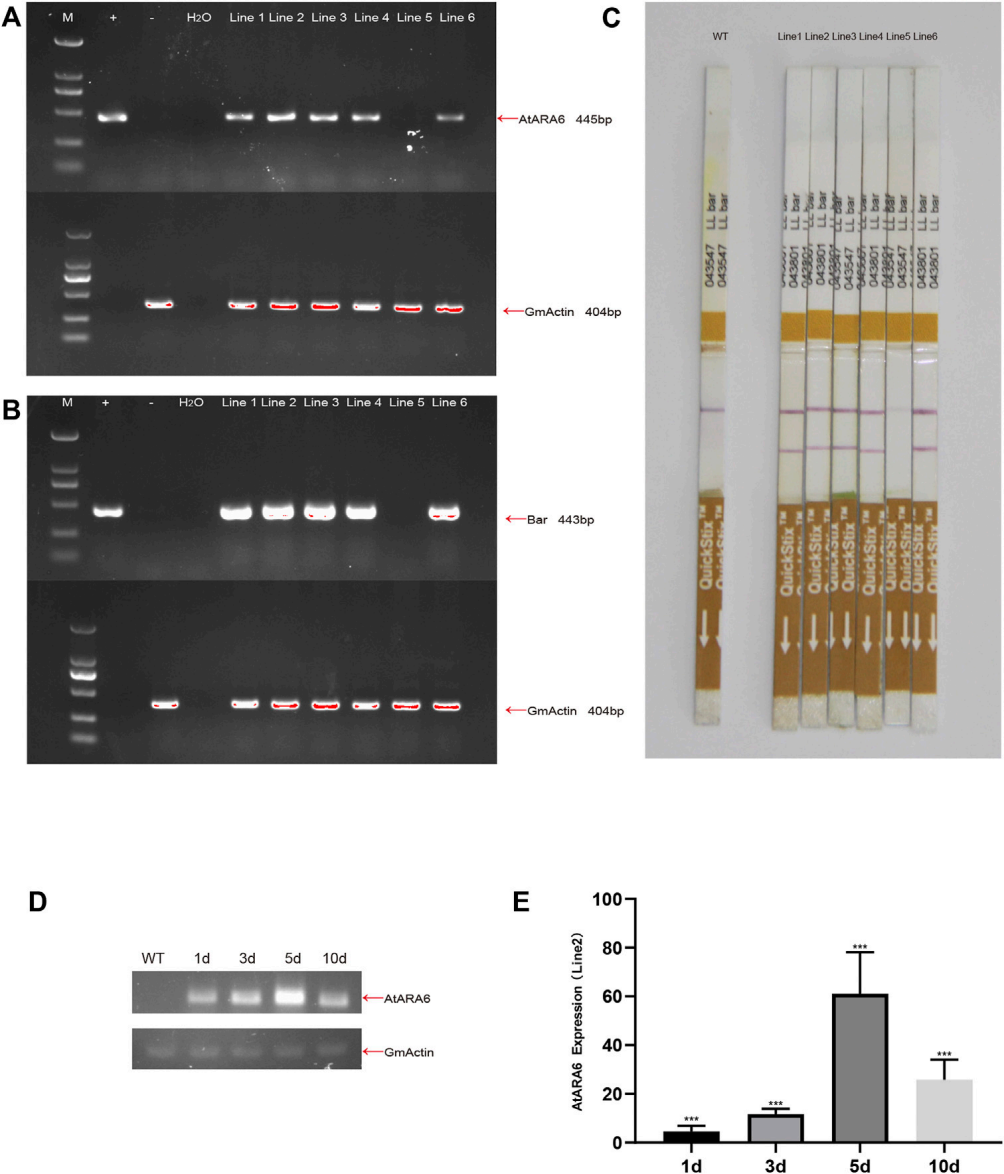
Confirmation of *AtARA6* transgenesis. **(A,B)** Positive transgenic soybean progeny Lines1-6: 404 bp of GmActin was used to detect plant genomic DNA, *AtARA6* 445 bp, *Bar* 443=bp (M, Trans2K Plus II DNA marker; +, *AtARA6* expression vector plasmid DNA; −, WT genomic DNA). **(C)** LibertyLink test strips for genetically transformed soybeans. **(D)** RT-PCR analysis of *AtARA6* gene expression at different times under 200 mM NaCl treatment. **(E)** qRT-PCR analysis of the relative expression of *AtARA6* in different durations under salt treatment.

Cultivated soybean, strain SN9, was transformed by agrobacterium harboring *AtARA6,* and a total of six transformation lines, named Line1, Line2, Line3, Line4, Line5, and Line6, were obtained in the T0 generation (Figure 1). PCR was used to detect the presence of *AtARA6* and *Bar* genes separately. Five positive transgenic lines were identified, consistent with the results of the LibertyLink test strips (Figures 2A–C). The positive lines were propagated to T3 to ensure stable transgene inheritance. qRT-PCR results demonstrated that *AtARA6* showed the highest expression level at 5 days after salt treatment (Figures 2D,E).

### *AtARA6* Gene Improves Soybean Salt Tolerance

**FIGURE 3:**
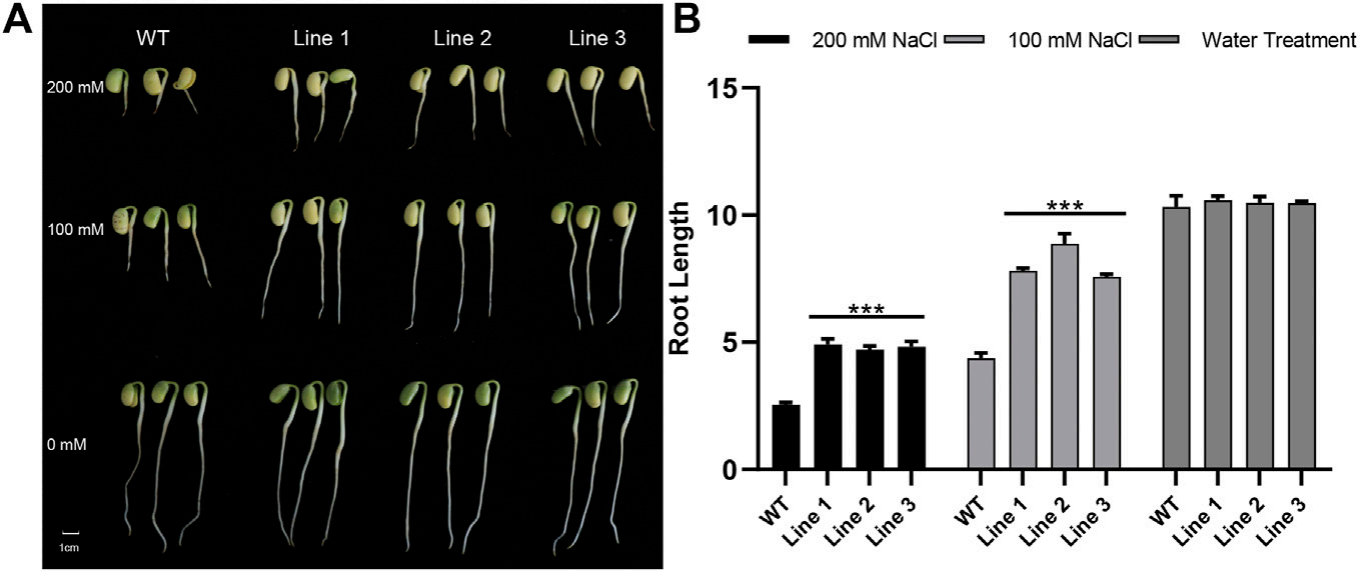
Salt treatment phenotypes of transgenic soybean and WT during germination. **(A)** Phenotypes of transgenic soybean Lines1-3 and WT treated with 200 mM NaCl, 100 mM NaCl, or water for 7 days at the germination stage. **(B)** Root length phenotypes of WT and transgenic soybean under salt stress during germination (*p* < 0.05).

We assessed the salt tolerance of transgenic soybean and WT plants in the germination and emergence stages separately. Three positive T3 generation transgenic lines and WT were sown in vermiculite treated with different concentrations of salt solution. The length of the roots was compared after 7 days of treatment. As shown in Figures 3A,B, there was no significant difference in root length between transgenic soybean and WT plants in the water treatment group. However, transgenic soybean roots were longer than WT in both 100 mM and 200 mM NaCl solutions (Supplementary Table S4), suggesting that transgenic soybean has stronger germination ability and adaptability under salt stress.

**FIGURE 4:**
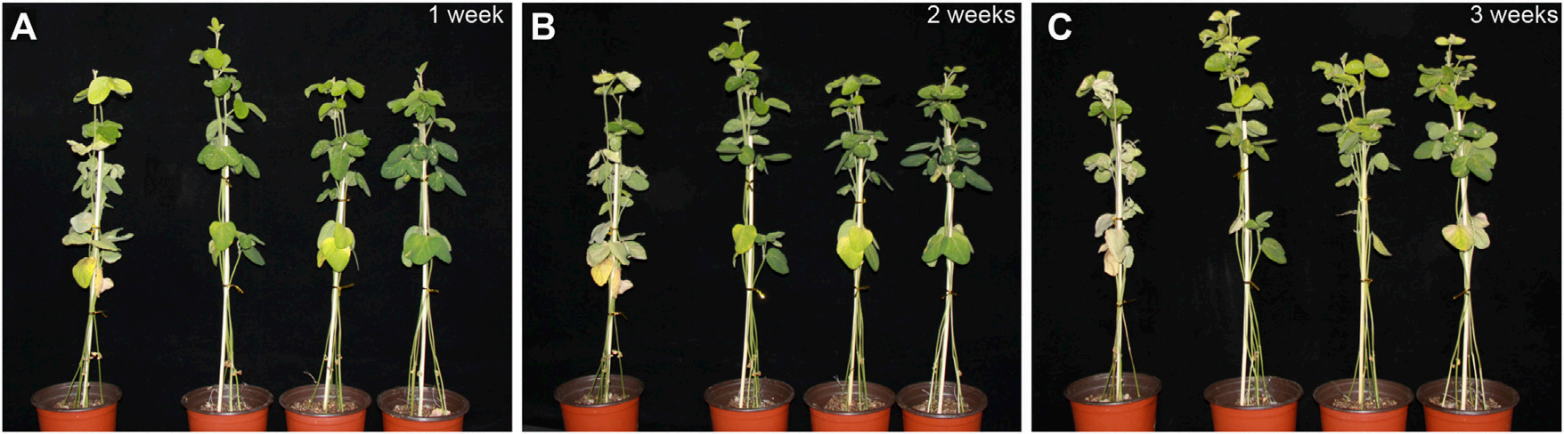
Salt treatment phenotypes of transgenic soybean and WT at emergence stages. **(A–C)** Phenotypes of transgenic soybean Lines1-3 and WT (left) treated with 200 mM NaCl at emergence stages for 1, 2, and 3 weeks.

We then treated transgenic soybean with unfolded opposite true leaves in 200 mM NaCl solution WT leaves started to turn yellow and dry at 1 week, and the true leaves wilted and fell off. There were no significant changes in the transgenic lines Line1 and Line3, although the true leaves of Line2 turned slightly yellow (Figure 4A). After 2 weeks of salt treatment, most of the lower leaves of WT plants lost their green color, appeared dehydrated, the petioles withered and fell off, the upper leaves turned yellow and appeared dehydrated, and only a few of the apical ternate leaves were unaffected. In contrast, only 1-2 true leaves of transgenic soybean Line1 and Line2 turned yellow, and the upper part of the plants stayed upright and grew normally (Figure 4B). After 3 weeks, the entire WT plant was withered, the growth point was necrotic, and the leaves were severely shed. The true leaves of Line1 and Line2 transgenic soybean did not fall off, and Line3 only had true leaves that turned yellow. The degree of salt damage in transgenic soybean was thus significantly lower than that in WT (Figure 4C). These results showed that the transformation using the *AtARA6* gene significantly improved the salt tolerance pathway of SN9 and enhanced soybean resistance to salt stress.

### Physiological Evaluation of Salt Tolerance

**FIGURE 5:**
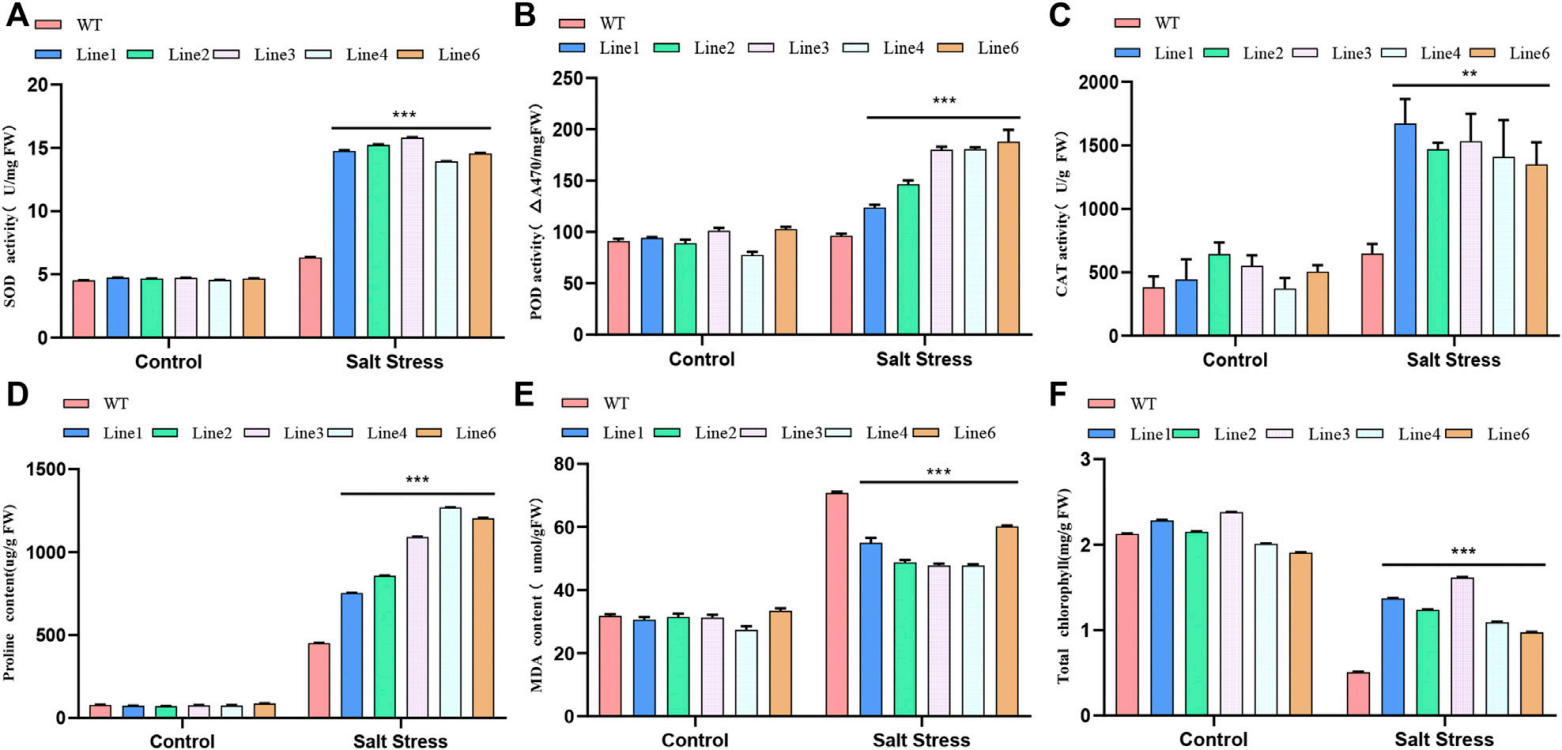
Determination of physiological parameters of transgenic soybean and WT treated with salt stress. **(A)** Superoxide dismutase (SOD) activity. **(B)** Peroxidase (POD) activity. **(C)** Catalase (CAT) activity. **(D)** Proline content in leaves (µg/g). **(E)** Malondialdehyde content in leaves (µmol/g). **(F)** Chlorophyll content of leaves after salt treatment (mg/g). All values were measured after 21 days of salt treatment. Statistical analysis was performed using one-way ANOVA, *p < 0.05, **p < 0.01, ***p < 0.001, *p* > 0.05 indicates the difference between transgenic line and WT was not significant.

To assess the salt tolerance level of transgenic soybean, we measured the SOD, POD, and CAT activities of transgenic soybean and WT plants under salt stress. All these activities in both transgenic soybean and WT increased in the 200 mM NaCl treatment group. However, the increase in these activities in transgenic soybean was significantly greater than that in WT, suggesting that transgenic soybean has a stronger tolerance capacity under salt stress (Figures 5A–C). Similarly, the increased proline content in the WT was much lower than that in the transgenic line (Figure 5D). MDA content reflects the degree of damage to plant membrane lipids. Both transgenic soybean and WT MDA contents increased under salt stress, but the transgenic line increased less than SN9 (Figure 5E). The chlorophyll content of the soybeans decreased under salt treatment, but that of the transgenic line was over 2-fold higher than that of the WT plants (Figure 5F). Under stress, the plant’s defense system responds by generating protective enzymes such as SOD, POD, and CAT, which can reduce ROS levels in cells. Proline, an osmoregulatory substance, can regulate the osmotic potential of plant cells. Protective enzyme activity and proline content increased in both transgenic soybean and WT under salt stress. However, the elevation was much higher in transgenic soybean than in WT. In addition, the transgenic soybean plasma membrane was less damaged than the WT, and chlorophyll levels were higher than in WT. Such differences suggest that *AtARA6* can aid soybean resistance to salt stress and reduce the degree of damage to the organism under such conditions.

### RNA-Seq Analysis of Transgenic Lines and WT

**FIGURE 6:**
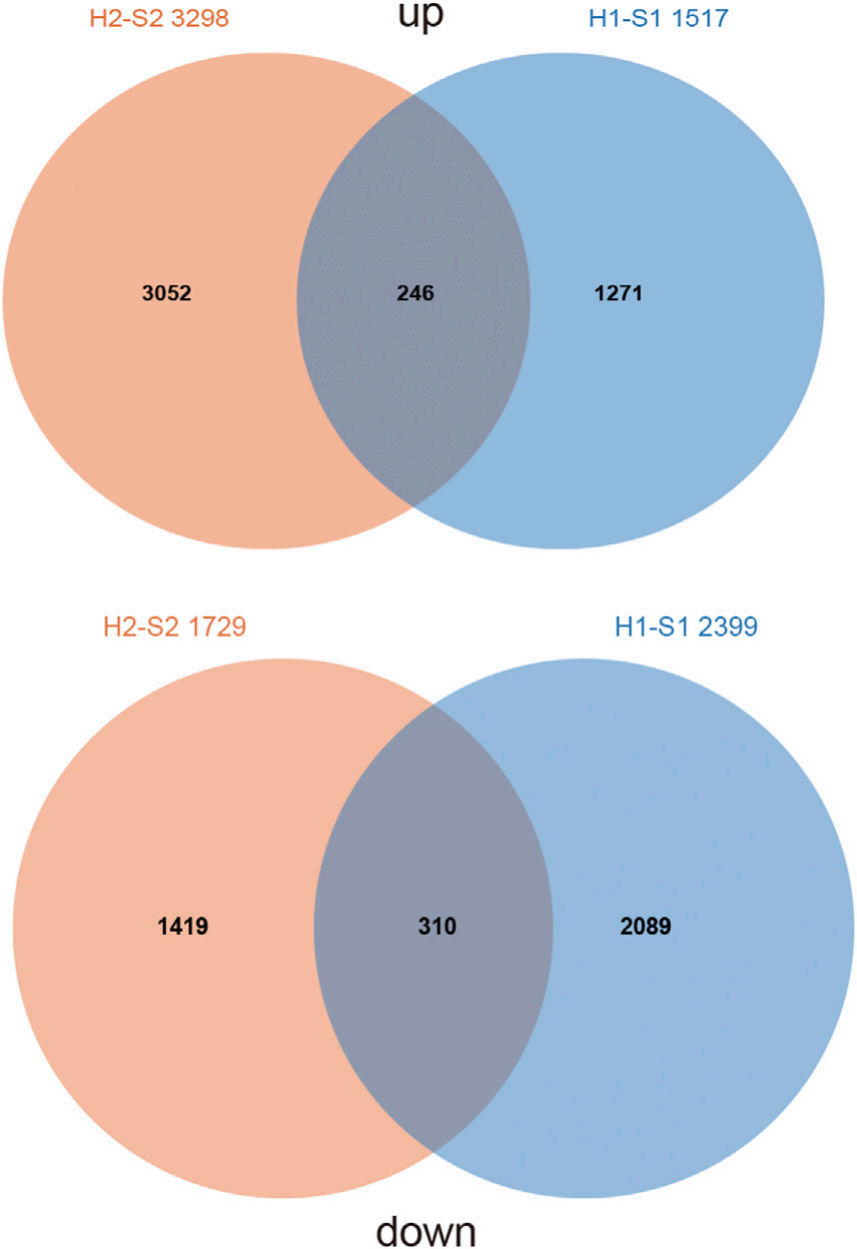
Comparison of DEGs between transgenic positive lines and SN9 under salt treatment and water treatment.

To investigate the potential pathways by which *AtARA6* affects salt tolerance, we performed RNA-seq analysis using Line2 and WT in the true leaf stage under salt stress for 5 days, named S1 and S2, respectively. The same plant materials were mock-treated with water, named H1 and H2. The clean data obtained from each of these samples was over 5.84 Gb, and the percentage of Q30 bases was above 92.13%. The clean data of each sample were compared with the Williams 82 reference genome separately, and the comparison efficiency ranged from 89.56% to 94.08% (Table 1). Between H1 and S1, we detected 3,916 DEGs, including 2,399 downregulated DEGs and 1,517 upregulated DEGs in transgenic soybean under salt treatment. In the H2 versus S2 group, we detected 5,027 DEGs, including 1,729 downregulated DEGs and 3,298 upregulated DEGs in SN9 under salt treatment (Figure 6). Furthermore, we compared the DEGs in the H2-S2 and H1-S1 groups, which shared only 246 upregulated DEGs (∼5.4%) and 310 downregulated DEGs (∼8.1%) (Figure 6). These results indicate that overexpression of the *AtARA6* gene in soybean resulted in significant alterations in gene transcription in SN9, which may be the reason for improved salt tolerance.

**TABLE 1:** Summary statistics of the RNA quality and sequencing results.

Clean data	59.68GB		
Q30	>92.13%		
Rades mapped rate	89.56%–94.08%		
All database annotation	57,265		
Clean data	59.68GB		
	Total reads on average	Mapped reads on average	Unmapped reads on average
H1	49,314,341	44,299,190 (89.81%)	5,015,151 (10.19%)
H2	48,795,122	45,559,462 (93.37%)	3,235,660 (6.64%)
S1	47,200,475	44,389,190 (94.05%)	2,811,286 (5.95%)
S2	54,559,953	51,243,100 (93.93%)	3,316,853 (6.08%)

### *AtARA6* and the Soybean SNARE Regulatory Pathway

**FIGURE 7:**
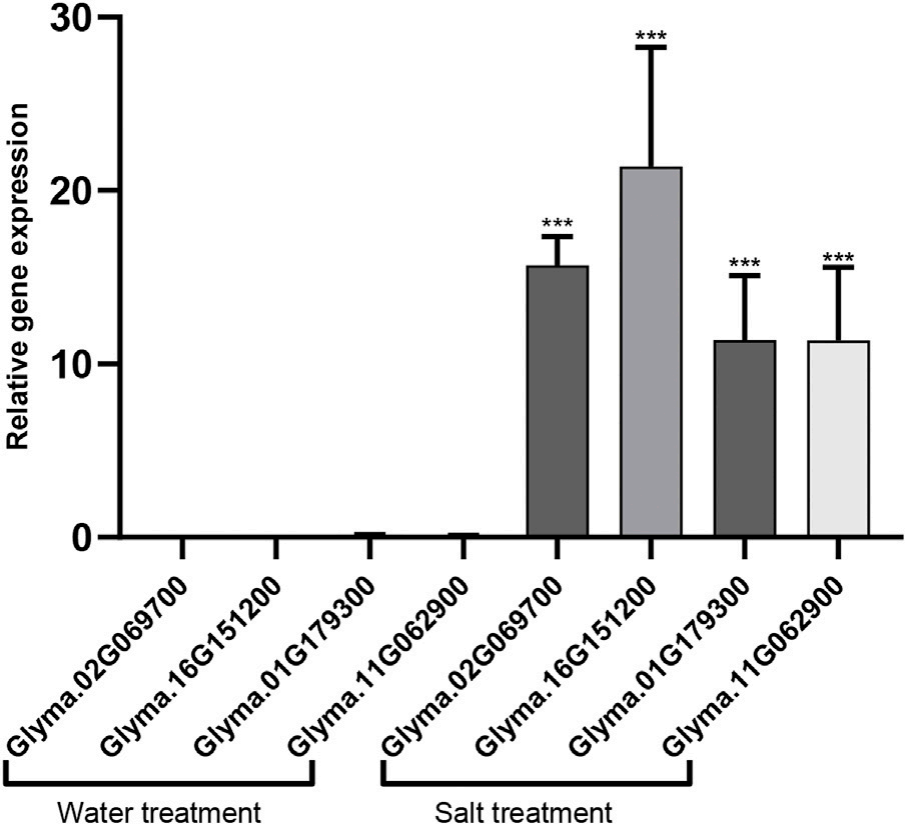
qRT_PCR analysis of homologous genes of *SYP121* and *VAMP727*. The homologous genes of *SYP121*, Glyma.02G069700 and Glyma.16G151200. The homologous genes of *VAMP727*, 01G179300 and Glyma.11G062900. They were significantly up-regulated under in transgenic soybean after salt treatment. Three biological replicates were used. Data was calculated using the 2^−ΔΔCT^ method, Statistical analysis was performed using ANOVA (*p* < 0.05), *p < 0.05, **p < 0.01, and ***p < 0.001.

In the vesicular transport pathway, SNARE complexes are responsible for the transport of substances from endosomes to the plasma membrane. *AtARA6* regulates the formation of SNARE complexes containing *VAMP727* and *SYP121* (Ebine et al., 2011). In S1 and S2 DEGs, we found two homologs of *SYP121* in soybean (Glyma.02G069700 and Glyma.16G151200). The KEGG results showed that they were significantly enriched in the SNARE pathway (Supplementary Table S5). *VAMP727* encodes a vesicle-associated membrane protein 727 on the target membrane and plays a role in the fusion of vesicles with the target membrane. We found two homologous genes of *VAMP727* in soybean (Glyma.01G179300 and Glyma.11G062900), which were significantly upregulated in transgenic soybean after salt treatment. KEGG enrichment results showed that these two genes are in the endocytosis pathway (Supplementary Table S5). We verified 4 upregulated genes using qRT-PCR (Figure 7). Our results show that *AtARA6* upregulates the SNARE genes *SYP121* and *VAMP727*.

### Gene Ontology Enrichment Analysis of Differentially Expressed Genes

To investigate the functions of DEGs, we performed GO analysis of DEGs in transgenic soybean and WT plants under salt and water treatments. We focused on 1,271 upregulated DEGs and 2,089 downregulated genes in the transgenic line under salt treatment (Figure 6). The expression of some of these genes may be perturbed by the *AtARA6* transgene. For 1,271 upregulated DEGs, significant GO terms in RNA-related processes were enriched, including RNA metabolic processes (GO:0016070, *p* = 1.10E-05), RNA biosynthetic processes (GO:0032774, *p* = 4.00E-05), regulation of transcription (GO:0006355, *p* = 1.10E-05), and regulation of gene expression (GO:0010468, *p* = 1.60E-05) (Table 2). We hypothesize that such upregulated RNA-related pathways may help to explain the stronger salt resistance of the transgenic line. It is well-known that plants use a wide array of mechanisms, including transcriptional regulation and posttranscriptional mechanisms, to survive under diverse stress conditions. At the post-transcriptional level, many studies have demonstrated that Ca^2+^-dependent transcriptional reprogramming is important for the stress response (Galon et al., 2008; Yuan et al., 2018a; Yuan et al., 2018b). In addition, RNA modification has been shown to be related to stress responses, such as splicing, capping, polyadenylation, and translocation. Overexpression of the Arabidopsis RNA binding protein *AtRGGA* enhances salt tolerance (Ambrosone et al., 2015). Similarly, overexpression of *Beta vulgaris* RNA binding protein, salt tolerant 3 (*BvSATO3*), in Arabidopsis increased plant salt resistance (Rosa Téllez et al., 2020).

**TABLE 2:** GO enrichment of 1,271 upregulated DEGs.

GO term	Ontology	Description	Genes	Total genes	*p*-value	FDR
GO:0016070	BP	RNA metabolic process	84	2,485	0.00021	0.0054
GO:0032774	BP	RNA biosynthetic process	80	2015	1.30E-06	4.00E-05
GO:0010468	BP	Regulation of gene expression	77	1850	3.80E-07	1.60E-05
GO:0006355	BP	Regulation of transcription, DNA-dependent	77	1796	1.20E-07	1.10E-05

For 2,089 downregulated DEGs in the transgenic line under salt treatment, significant GO terms in metabolism-related processes, (GO:0008152, *p* = 1.50E-27), primary metabolic processes (GO:0044238, *p* = 0.0037), cellular metabolism (GO:0044237, *p* = 2.50E-08), macromolecule metabolism (GO:0043170, *p* = 0.019), protein metabolism (GO:0019538, *p* = 0.0019), carbohydrate metabolism (GO:0044262, *p* = 0.00077), and glucose metabolism (GO:0006006, *p* = 1.60E-05) (Table 3). We believe that the reduction of the expression of certain metabolism-related genes may indirectly reduce the pressure to maintain cellular homeostasis, which is perturbed by salt stress. A similar result was found for gene expression changes in the sugar beet under salt stress (Rosa Téllez et al., 2020).

**TABLE 3:** GO enrichment of 2089 downregulated DEGs.

GO term	Ontology	Description	Genes	Total genes	*p*-value	FDR
GO:0006066	BP	Alcohol metabolic process	17	24	2.10E-12	3.40E-10
GO:0043170	BP	Macromolecule metabolic process	427	7,301	0.00075	0.019
GO:0044238	BP	Primary metabolic process	529	9,070	0.00013	0.0037
GO:0019538	BP	Protein metabolic process	281	4,388	4.30E-05	0.0019
GO:0044262	BP	Cellular carbohydrate metabolic process	30	235	1.60E-05	0.00077
GO:0044237	BP	Cellular metabolic process	521	8,043	2.20E-10	2.50E-08
GO:0008152	BP	Metabolic process	842	12,346	2.70E-30	1.50E-27

**FIGURE 8:**
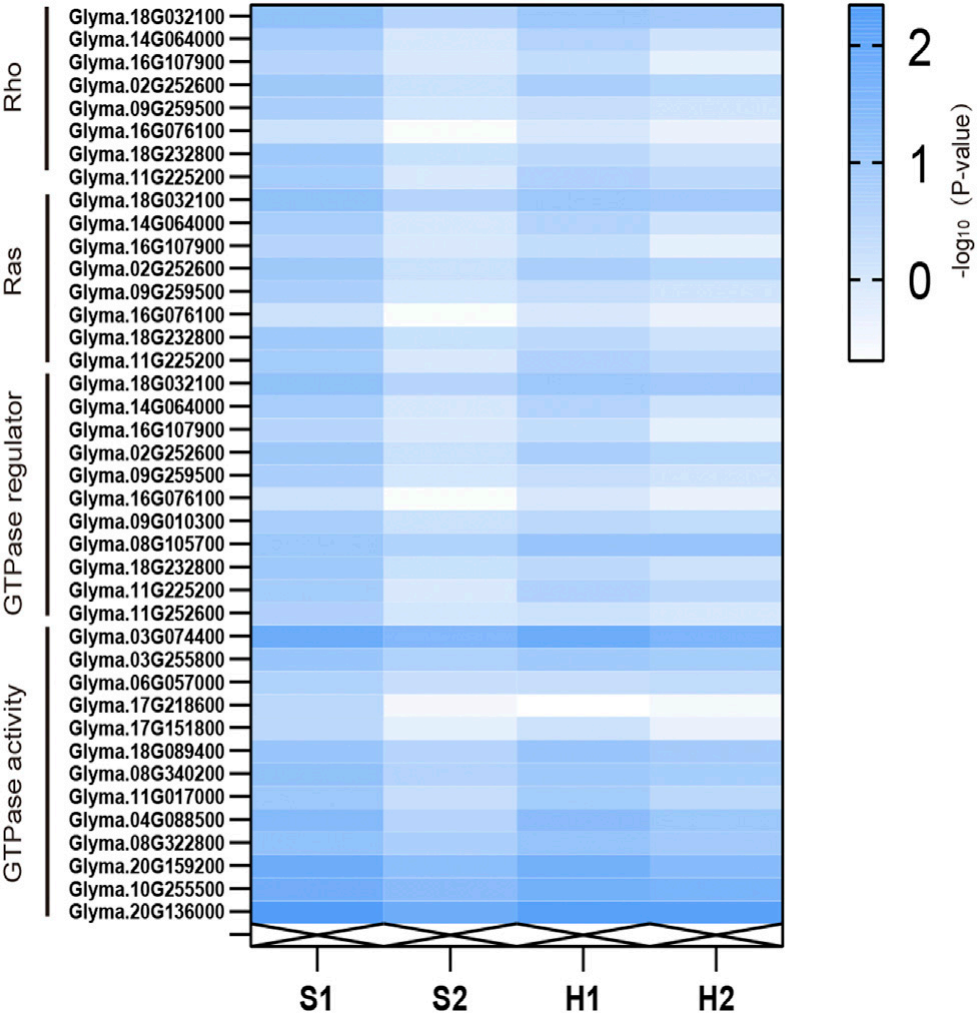
RNA-seq reveals the expression levels of DEGs enriched in the Rho/Ras/GTPase regulator/GTPase activity pathways under different treatments.

In addition, we analyzed the direct mechanism via which *AtARA6* improves salt tolerance in soybean. Between the S1 and S2 groups, we identified 6,572 DEGs that represent unique processes carried out by transgenic soybean under salt treatment. Among the GO terms, Rho guanyl-nucleotide exchange factor activity (GO:0005089, *p* = 0.0045), Ras guanyl-nucleotide exchange factor activity (GO:0005088, *p* = 0.0045), GTPase regulator activity (GO:0030695, *p* = 0.0093), and GTPase activity (GO:0003924, *p* = 0.044) pathways were significantly enriched in the molecular function module in upregulated DEGs in transgenic soybean under salt treatment (Supplementary Figure S2). Rho-like GTPases are plant-specific molecular switches that play a critical role in plant survival under abiotic stresses (Miao et al., 2018). The expression levels of DEGs enriched in these pathways under different treatments were found to be higher in transgenic soybean than in WT (Figure 8). Therefore, we suggest that the upregulation of these four pathways by the *AtARA6* gene acted as a direct factor to improve the salt tolerance of transgenic soybean.

**FIGURE 9:**
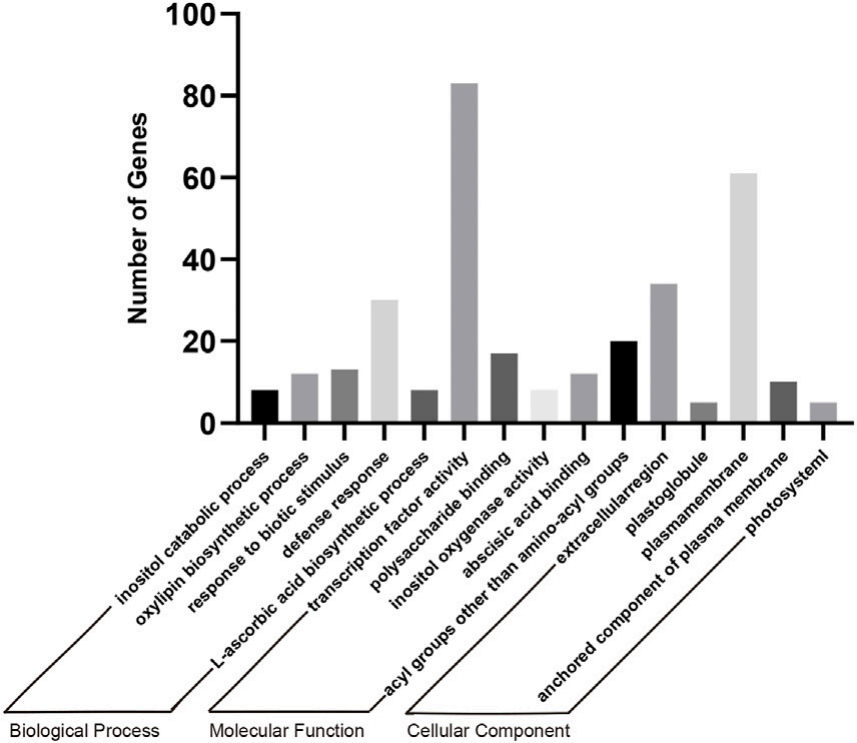
GO enrichment analysis of 935 DEGs including BP, CC and MF. *p*-values < 0.01 for all GOs. Transcription factor activity, sequence-specific DNA binding (GO:0003700, *p* = 5.2e-11, MF) and inositol catabolic process (GO:0019310, *p* = 4.0e-10, BP) are the most significant.

After the GO analysis of DEGs between different treatments, we concluded that the transgenic soybean showed significant enrichment in terms of transcription level and substance metabolism. In order to explore the changes caused by the transformation of *AtARA6* in soybean, we combined S1, S2 and H1, H2 for analysis. We first found 3,815 DEGs between H1 vs. S1 and 4,933 DEGs between H2 vs. S2. Among these DEGs, 1,344 DEGs were shared and could be considered as changes in soybean caused by salt stress. At the same time, we identified 6,456 DEGs between S1 vs. S2 as a result of transfection of the *AtARA6* gene. We found 935 DEGs between 1,344 and 6,456 DEGs, which represented the unique effects of *AtARA6* gene transformation under salt stress (Supplementary Figure S3). GO analysis of 935 DEGs showed that they had the highest levels of transcription factor activity, sequence-specific DNA binding (GO:0003700, *p* = 5.2e-11, MF), and inositol catabolic process (GO:0019310, *p* = 4.0e-10, BP), and the difference was significant (Figure 9). Furthermore, we identified multiple salt stress-responsive transcription factors. *MYC2* is a negative regulator of proline synthesis and improves salt tolerance by regulating the proline content in Arabidopsis (Verma et al., 2020). In addition, *WRKY6* and *WRKY86* have been reported to negatively regulate plant salt tolerance (Li et al., 2019; Fang et al., 2021). In our RNA-seq results, the expression levels of *MYC2*, *WRKY6*, and *WRKY86* were all downregulated (Supplementary Table S5), indicating that they are involved in the salt tolerance pathway of transgenic soybean. Moreover, the phospholipase signaling pathway is an important pathway for plant salt tolerance. The N-terminus of phospholipase D contains a domain that binds to phosphoinositide. At the same time, *PLDα* can bind to the heterotrimeric protein subunit to regulate GTPase activity and control stomatal movement and loss of water (Zhao and Wang, 2004). GO results showed that transgenic soybeans were significantly enriched in the inositol catabolic process and inositol oxygenase activity (GO:0050113, *p* = 2.7e-9) pathways. Four DEGs were found to be enriched in this pathway (Glyma.08G199300, Glyma.07G126600, Glyma.05G224500, and Glyma.07G013900), all of which functioned to encode inositol oxygenase. In rice, inositol oxygenase has been reported to scavenge reactive oxygen species (Duan et al., 2012). In our study, these four genes were strongly upregulated after transgenic soybean salt treatment, indicating that transgenic soybeans could regulate the inositol catabolic process to improve salt tolerance.

### Kyoto Encyclopedia of Genes and Genomes Pathway Enrichment Analysis of Differentially Expressed Genes

**FIGURE 10:**
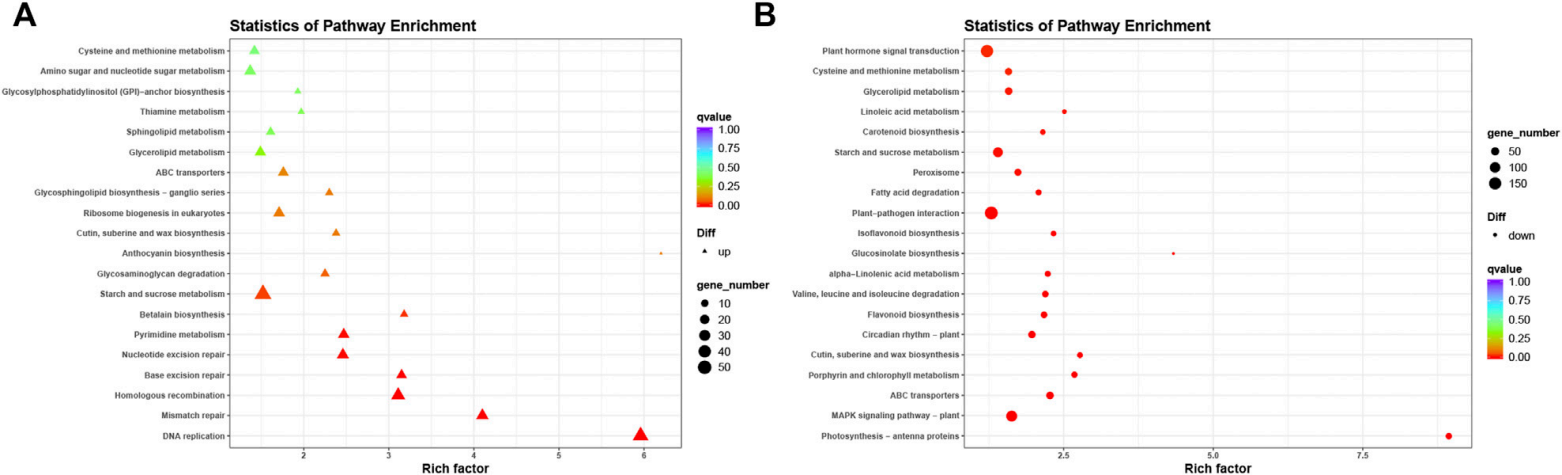
Kyoto Encyclopedia of Genes and Genomes analysis of up-regulation and down-regulation of DEGs. **(A,B)** KEGG analysis on up-regulated (left) and down-regulated (right) DEGs under salt stress in transgenic soybean compared to WT.

To further investigate the mechanisms for the increased salt tolerance of transgenic soybean, we performed KEGG analysis on up and downregulated DEGs under salt stress in transgenic soybean compared to WT (Figure 10A). Among the upregulated DEGs, there was enrichment in DNA replication (ko03030, *p* = 6.04e-80), mismatch repair (ko03430, *p* = 7.22e-25), pyrimidine metabolism (ko00240, *p* = 5.26e-43), homologous recombination (ko03440, *p* = 8.41e-32), base excision repair (ko03410, *p* = 1.17e-26), nucleotide excision repair (ko03420, *p* = 7.24e-32), and starch and sucrose metabolism (ko00500, *p* = 1.68e-98) (Supplementary Table S7). Previous studies have shown that salt stress disrupts genome stability in plants (Roy et al., 2013). Exposure to high salinity increases the risk of DNA double-strand breaks (Roy et al., 2013). Our transgenic soybean showed upregulated genes related to DNA replication and mismatch repair pathways, possibly partially underlying its stronger ability to cope with salt stress.

In the downregulated DEGs, significantly enriched pathways included photosynthesis-antenna proteins (ko00196, *p* = 4.53e-51), porphyrin and chlorophyll metabolism (ko00860, *p* = 2.64e-41), cutin suberine and wax biosynthesis (ko00073, *p* = 1.10e-48), flavonoid biosynthesis (ko00941, *p* = 2.80e-43), cysteine and methionine metabolism (ko00270, *p* = 8.57e-88), and linoleic acid metabolism (ko00591, *p* = 1.84e-37; Figure 10B) (Supplementary Table S8). Many downregulated pathways were concentrated in biosynthesis and metabolism processes, which were in line with our hypothesis based on GO analysis that reduction of certain metabolism-related gene expression may indirectly reduce the pressure to maintain cellular homeostasis, which is perturbed by salt stress. The downregulated pathways in our study were consistent with those of previous studies. For example, cysteine and methionine levels decreased significantly in broccoli after salt treatment (Lopez-Berenguer et al., 2008). In peanuts, linoleic acid synthesis genes were also downregulated after salt treatment (Sui et al., 2018).

## Discussion

Salt stress is an important factor that affects soybean yield and growth. Transgene breeding has proven to be a promising method for obtaining salt-tolerant cultivars. For example, soybean *GmNHX1* can accelerate the transport of Na^+^ from the cytoplasm to the vesicles. Salt tolerance was improved in both soybean and Arabidopsis by overexpression of *GmNHX1* (Sun et al., 2019). In rice, overexpression of *OsRab7* increases endocytosis and leads to increased salt tolerance (Peng et al., 2014).

In this study, an Arabidopsis key vesicle trafficking gene, *AtARA6*, was transformed into soybean and stable expression lines were confirmed by PCR and qRT-PCR. Some stress-related indicators, such as protective enzymes, proline, and MDA, were found to be upregulated, perhaps underlying the ability of transgenic soybean to withstand adversity. Our results showed that the transgenic soybean grew normally at the germination and emergence stages under salt treatment, compared to WT, which showed growth stagnation and eventual death.

*AtARA6* belongs to the RAB5 family. Its N-terminal myristoylation and palmitoylation sites distinguish it from other RAB5 members. The relationship between these sites and salt tolerance has been demonstrated in both *AtARA6* and other genes. For example, overexpression *of AtARA6* in Arabidopsis under salt treatment yields longer roots compared to WT, indicating stronger salt resistance. However, overexpression of *AtARA6* with a 29 amino acid truncation at the N-terminus shows a similar root length to WT, indicating that the N-terminus is important for salt resistance (Yin et al., 2017). Similarly, the Arabidopsis *AtSOS3* gene plays a role in the salt response. The rescue of the salt-hypersensitive phenotype of *AtSOS3-1* knockout plants required the full-length *AtSOS3* gene, but the *AtSOS3* gene with an N-terminal mutant did not rescue the phenotype (Ishitani et al., 2000).

**FIGURE 11:**
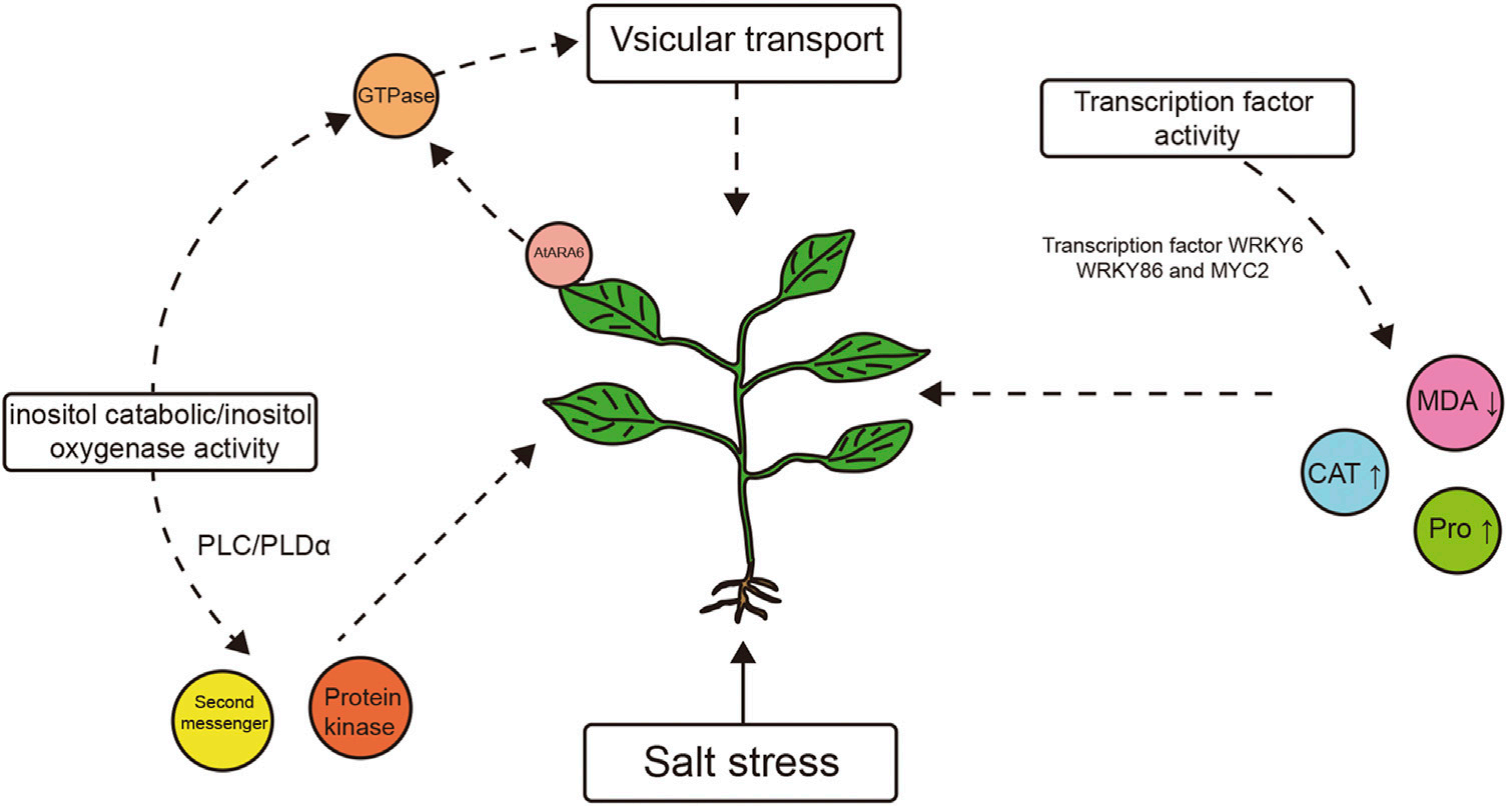
Salt tolerance pathway of *AtARA6* transgenic soybean.

In Arabidopsis, SNARE is a pathway in which *AtARA6* is directly involved, and *AtARA6* has been shown to enhance salt tolerance in Arabidopsis through vesicular transport. In this study, we focused on *SYP121* and *VAMP727* genes. qRT-PCR results showed that their expression was upregulated under salt treatment, more in transgenic plants than in WT plants, suggesting that the transformation of the *AtARA6* gene accelerated vesicle movement in soybean and that the improvement in salt tolerance in soybean is due to this, as it is in Arabidopsis. Many functions of *AtARA6* in Arabidopsis have been reported: for example, the *AtARA6* overexpression line is more tolerant to dark-induced senescence (DIS) (Yin et al., 2017). Recent studies have shown that *AtARA6* can affect the expression of the starch gene *QQS* in Arabidopsis, thus participating in the dynamic balance between starch and soluble sugars in Arabidopsis. A comparison of wild-type and *AtARA6* mutants revealed that *AtARA6* inhibited the proliferation of pathogenic bacteria (Tsutsui et al., 2015). We explored the function of *AtARA6* in transgenic soybeans. Here, we performed RNA-seq on transgenic soybean and SN9 under different treatments using leaves but not roots. Although leaves are not the first organ to respond to salt stress, considering the function of *AtARA6*, it has been reported that Qc-SNAREs transfer excess Na^+^ to vacuoles, thus increasing salt tolerance in Arabidopsis (Liu et al., 2007). As the vacuoles in the primary roots were immature, leaves became the first choice. In addition, plants can also regulate body water through the opening and closing of stomata. *SYP121* and *VAMP71* have been reported to be related to stomata closure (Grefen et al., 2015; Salinas-Cornejo et al., 2019). Leaves contain a larger number of stomata compared to roots; hence, they may be an important factor affecting the salt tolerance of soybeans. We identified significant enrichment of RNA-related processes and metabolic processes such as protein metabolism (GO:0019538), suggesting that transgenic plants can adapt to high salt stress through transcriptional regulation and metabolic regulation. At the same time, we comprehensively analyzed the DEGs of transgenic soybean and SN9 under different treatments and found that inositol catabolic process and transcription factor activity and sequence-specific DNA binding were significantly enriched in transgenic soybean under stress conditions, indicating that the two pathways are used for salt tolerance improvement. We have summarized the salt tolerance pathway of the *AtARA6* transgenic soybean in a map (Figure 11). Reactive oxygen species scavenging mechanisms are one of the important pathways for plants to cope with abiotic stress. In our study, four genes encoding inositol oxygenases were found to be significantly upregulated in transgenic soybeans after salt treatment, which was consistent with our physiological indicator measurements. In addition, we identified multiple transcription factors that respond to salt stress, which can serve as candidate genes for studying salt tolerance in transgenic soybeans. Under drought and salt stress, *WRKY6* negatively regulates the ability of Arabidopsis and cotton to tolerate abiotic stress; *WRKY86* can bind to maize W-box, thereby increasing maize catalase activity, reducing malondialdehyde content, and improving salt tolerance. Moreover, the transcription factor *MYC2* of the bHLH family enhances salt tolerance in Arabidopsis by regulating proline content. They provided the basis for the changes in physiological indexes of transgenic soybean under salt stress. These findings suggest that transgenic soybeans have salt tolerance pathways other than the SNARE pathway, in contrast to Arabidopsis.

Meanwhile, terms like Rho guanyl-nucleotide exchange factor activity, Ras guanyl-nucleotide exchange factor activity, GTPase regulator activity, and GTPase activity were found to be significantly enriched. To demonstrate that the enrichment of these pathways is not caused by *AtARA6* homologous genes in soybean, we investigated the expression of five *AtARA6* homologous genes. It was found that the expression levels of these five genes were not significantly different between SN9 and transgenic soybean under salt treatments, indicating that the upregulation of these pathways is caused by the *AtARA6* gene itself.

Sometimes, plants need to cope with diverse stress threats simultaneously. On one hand, combined stress conditions require a trade-off between stress adaptation and growth. Starch and sucrose metabolism was one of the most significant upregulated terms in our KEGG analysis. Starch has been proved to be a key player in response to water deficit, high salinity, and extreme temperatures. Under these adverse environmental conditions, chloroplasts and photosynthesis may be compromised, as shown in our study. Starch metabolism and remobilization provide extra energy and carbon for the plant to survive. The correlation between starch metabolism and diverse stress resistance has been widely proved. For example, in the model plant, Arabidopsis, salt stress induces rapid carbohydrate metabolism and increases soluble sugars (Kempa et al., 2008). Similarly, in sugar beet roots, upregulated starch and sucrose metabolism was found by RNA-seq of the plants with higher salt resistance levels (Liu et al., 2020).

Previous studies have shown that salt stress affects genome stability and induces DNA double-strand breaks in plants (Roy et al., 2013). The notable enriched upregulated KEGG terms in our transgenic soybean, mismatch repair and DNA replication, may be associated with salt tolerance by maintaining genome stability. Although the connection is not fully understood, some evidence has linked genome stability and stress resistance. For example, the eukaryotic pre-replicative complex (PreRC), including heterohexameric minichromosome maintenance proteins (MCM), is responsible for making sure the DNA is replicated only once per cell division cycle. Overexpression of a MCM family member, *MCM6*, in tobacco confers higher salinity tolerance (Tuteja et al., 2011). Moreover, topoisomerase is the specific enzyme that can remove or add DNA supercoils and untangle snarled DNA. Enhanced salt tolerance was found in transgenic tobacco overexpressing *NtTopoIIα-1*, a topoisomerase gene (John et al., 2016).

On the other hand, prioritization of different stress adaptations is also critical. In Arabidopsis, salt treatment induces decreased resistance to pathogens such as *Pseudomonas syringae*, *Alternaria brassicicola*, and *Botrytis cinerea* (Haller et al., 2020). Similarly, Matthias et al. found that ABA, a stress plant hormone, can repress immunity against a bacterial strain, *Pto hrcC−*, in old leaves (Berens et al., 2019). In cotton, overexpression of the *GhMKK1* gene enhances salt tolerance, as well as the susceptibility to the pathogen Ralstonia solanacearum (Lu et al., 2013). In our study, we found overexpression of an Arabidopsis gene, AtARA6, enhances soybean salt resistance. Through KEGG analysis, we found that downregulated genes were significantly enriched in plant-pathogen pathways. Our data suggest that heterologous expression of the AtARA6 gene in soybean may prioritize the salt tolerance response over the pathogen response.

## Data Availability

The datasets presented in this study can be found in online repositories. The names of the repository/repositories and accession number(s) can be found below: https://www.ncbi.nlm.nih.gov/, PRJNA789350.
