# Erratum: Heterologous expression of Arabidopsis *AtARA6* in soybean enhances salt tolerance

**DOI:** 10.3389/fgene.2022.1017834

**Published:** 2022-09-08

**Authors:** Frontiers Production Office

**Keywords:** soybean, *AtARA6*, salt tolerance, RAB GTPase, RAB5, SNARE pathway

Due to a production error, there was an error in the Affiliation of Author Yang Li.

Instead of “Affiliation 2: Cultural Creativity and Management School, Communication University of Zhejiang, Hangzhou, China,” it should be “Affiliation 1: Key Laboratory of Soybean Biology in Chinese Ministry of Education, Northeast Agricultural University, Harbin, China.“ This has been corrected and all the remaining affiliations have been reordered.

“Zhipeng Hong^1†^, Yang Li^1†^, Yang Zhao^1†^, Mingyu Yang^1^, Xiaoming Zhang^1^, Yuhan Teng^1^, Linjie Jing^1^, Danxun Kong^1^, Tongxin Liu^1^, Shuanglin Li^1^, Fanli Meng^1^*, Qi Wang^2^* and Ling Zhang^3^* ^1^Key Laboratory of Soybean Biology in Chinese Ministry of Education, Northeast Agricultural University, Harbin, China, ^2^Institute of Crop Cultivation and Tillage, Heilongjiang Academy of Agricultural Sciences, Harbin, China, ^3^Agro-Biotechnology Research Institute, Jilin Academy of Agricultural Sciences, Changchun, China.”

Also, there was a typo in one of the Keywords. “sybean” has been corrected to “soybean.”

The publisher apologizes for this mistake. The original version of this article has been updated.

