# Supplementary material for: Heterologous Expression of Arabidopsis AtARA6 in Soybean Enhances Salt Tolerance
Source: Front Genet. 2022 May 12;13:849357. doi: 10.3389/fgene.2022.849357 (PMC9134241; doi:10.3389/fgene.2022.849357)
Supplement: Supplementary file 1 [file Table1.docx]

**Supplementary Table 1_ PCR detection primers**

| gene | forward primer | reverse primer |
| --- | --- | --- |
| AtARA6 | ATGAAGAACATGCCGTTC | CAGCTGATGCCAAAAATC |
| Bar | CAGCTGCCAGAAACCCACGTCAT | GCACCATCGTCAACCACTACATCGA |
| GmActin | TTGACTGAGCGTGGTTATTCC | GATCTTCATGCTGCTGGGTG |
|  |  |  |
