# Supplementary material for: Heterologous Expression of Arabidopsis AtARA6 in Soybean Enhances Salt Tolerance
Source: Front Genet. 2022 May 12;13:849357. doi: 10.3389/fgene.2022.849357 (PMC9134241; doi:10.3389/fgene.2022.849357)
Supplement: Supplementary file 2 [file Table8.docx]

**Supplementary Table 8_** **Significant KEGG pathways of downregulated DEGs**

| **#Term** | **Database** | **ID** | **Input** | **Total** | **P-Value** | **Corrected P-Value** |
| --- | --- | --- | --- | --- | --- | --- |
| Photosynthesis - antenna prote... | KEGG PATHWAY | ko00196 | 17 | 30 | 4.53E-51 | 9.06E-51 |
| Porphyrin and chlorophyll meta... | KEGG PATHWAY | ko00860 | 18 | 80 | 8.16E-42 | 2.45E-41 |
| Cutin, suberine and wax biosyn... | KEGG PATHWAY | ko00073 | 14 | 44 | 5.76E-35 | 2.3E-34 |
| Flavonoid biosynthesis | KEGG PATHWAY | ko00941 | 17 | 91 | 1.18E-39 | 8.27E-39 |
| Cysteine and methionine metabo... | KEGG PATHWAY | ko00270 | 29 | 179 | 2.58E-66 | 7.22E-65 |
| Linoleic acid metabolism | KEGG PATHWAY | ko00591 | 11 | 46 | 6.74E-32 | 6.07E-31 |
