# Supplementary figures and images for: Heterologous Expression of Arabidopsis AtARA6 in Soybean Enhances Salt Tolerance
Source: Front Genet. 2022 May 12;13:849357. doi: 10.3389/fgene.2022.849357 (PMC9134241; doi:10.3389/fgene.2022.849357)

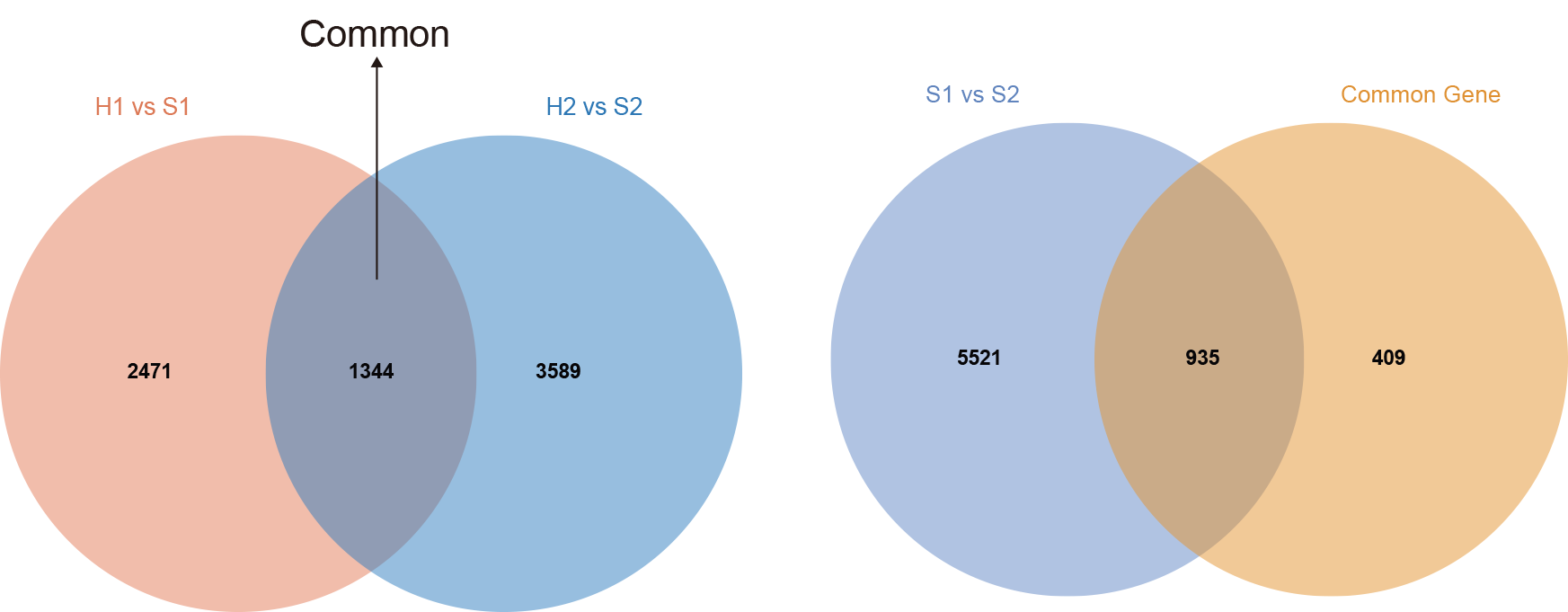

Supplement: Supplementary file 3 [file Image3.tif]

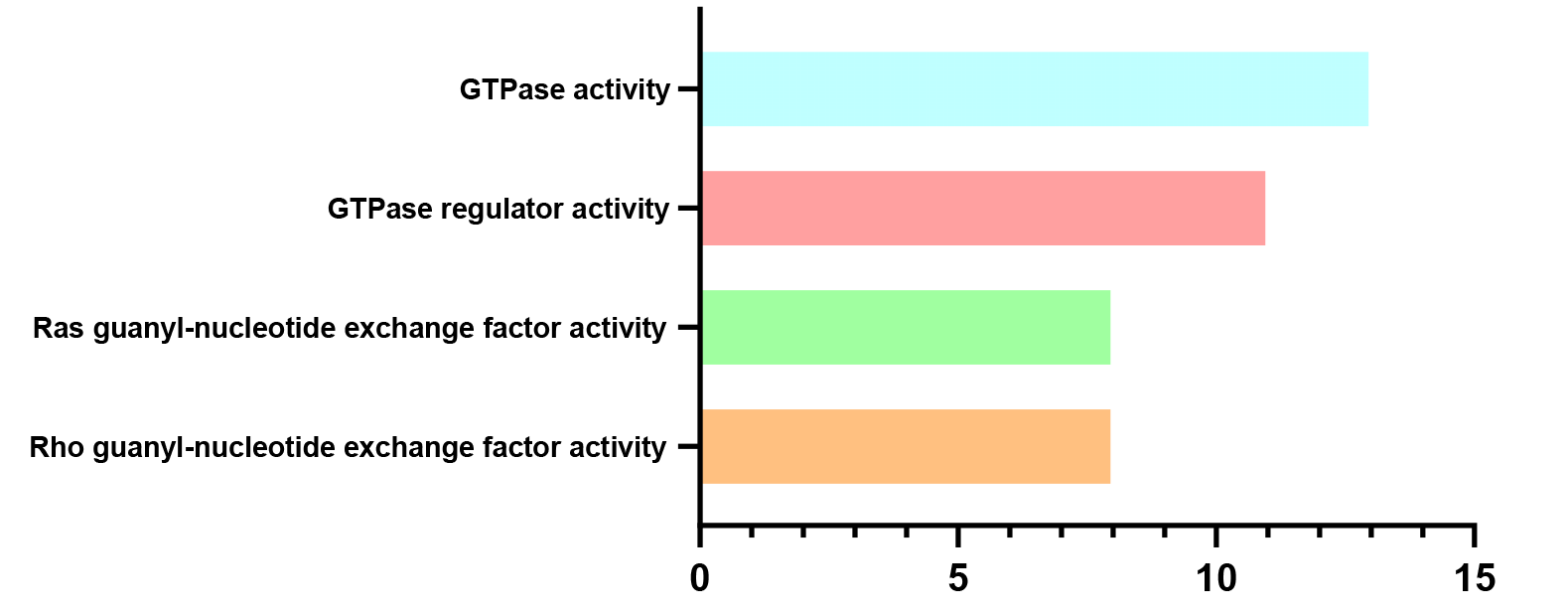

Supplement: Supplementary file 4 [file Image2.tif]

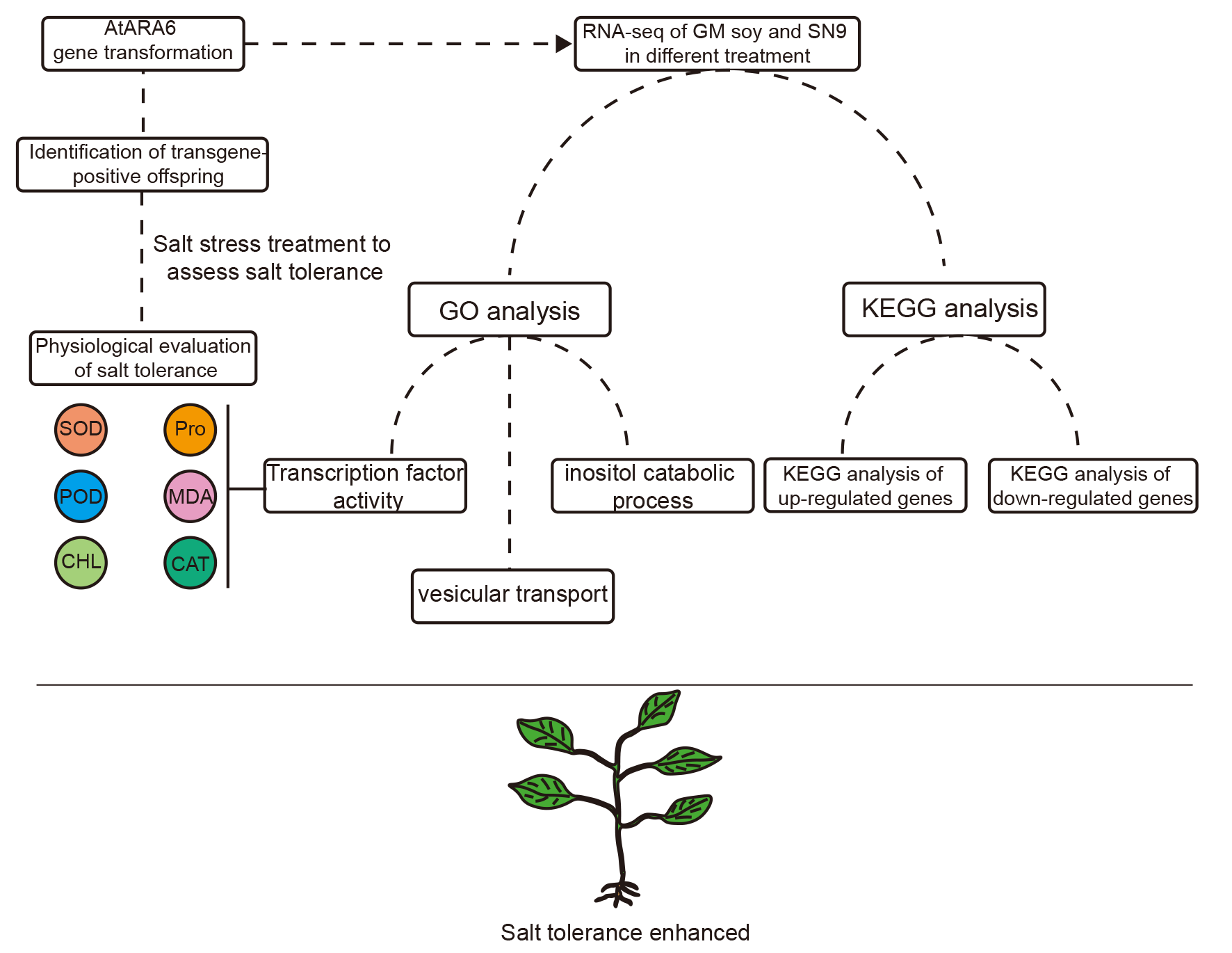

Supplement: Supplementary file 6 [file Image1.tif]
