# Supplementary material for: Heterologous Expression of Arabidopsis AtARA6 in Soybean Enhances Salt Tolerance
Source: Front Genet. 2022 May 12;13:849357. doi: 10.3389/fgene.2022.849357 (PMC9134241; doi:10.3389/fgene.2022.849357)
Supplement: Supplementary file 5 [file Table7.docx]

**Supplementary Table 7_** **Significant KEGG pathways of upregulated DEGs**

| **#Term** | **Database** | **ID** | **Input** | **Total** | **P-Value** | **Corrected P-Value** |
| --- | --- | --- | --- | --- | --- | --- |
| DNA replication | KEGG PATHWAY | ko03030 | 17 | 32 | 5.19E-81 | 2.60E-80 |
| Mismatch repair | KEGG PATHWAY | ko03430 | 18 | 12 | 4.75E-25 | 9.26E-25 |
| Nucleotide excision repair | KEGG PATHWAY | ko03420 | 14 | 13 | 5.56E-25 | 9.26E-25 |
| Homologous recombination | KEGG PATHWAY | ko03440 | 17 | 8 | 1.00E-14 | 1.25E-14 |
| Base excision repair | KEGG PATHWAY | ko03410 | 29 | 5 | 2.89E-09 | 2.89E-09 |
| Pyrimidine metabolism | KEGG PATHWAY | ko00240 | 11 | 14 | 4.51E-34 | 2.25E-33 |
