# Supplementary material for: Heterologous Expression of Arabidopsis AtARA6 in Soybean Enhances Salt Tolerance
Source: Front Genet. 2022 May 12;13:849357. doi: 10.3389/fgene.2022.849357 (PMC9134241; doi:10.3389/fgene.2022.849357)
Supplement: Supplementary file 8 [file Table2.docx]

**Supplementary Table 2_qRT_PCR primers**

| gene | forward primer | reverse primer |
| --- | --- | --- |
| Glyma.02G069700 | TCAGAAGCCGAATCAACGCT | CAATCCGGGTCCGGTAGATG |
| Glyma.16G151200  Glyma.01G179300  Glyma.11G062900 | ACCCTTTTGGTTCTTTCC  TCTTCTCTCGTTCTTGGC  TTCAGCAAAATGGGAGAC | TCAGCCTTCACCTTCTCA  ATGATGGGGGCGTTC  GAAGCAGCAGCAAAGG |
| Actin-1 | TTGACTGAGCGTGGTTATTCC | GATCTTCATGCTGCTGGGTG |
| Actin-2 | TGGTGCTGCCGCTATTTACTG | GGTGGAAGGAACTGCTAACAATC |
