# Supplementary material for: Heterologous Expression of Arabidopsis AtARA6 in Soybean Enhances Salt Tolerance
Source: Front Genet. 2022 May 12;13:849357. doi: 10.3389/fgene.2022.849357 (PMC9134241; doi:10.3389/fgene.2022.849357)
Supplement: Supplementary file 9 [file Table5.docx]

**Supplementary Table 5_Four homologous genes KEGG enrichment pathways**

| Gene | Pathway | ko_ID | DEgene | Number of genes | FDR | log2FC | regulated |
| --- | --- | --- | --- | --- | --- | --- | --- |
| Glyma.02G069700.Wm82.a2.v1 | SNARE interactions in vesicular transport | ko04130 | 2 | 103 | 1.52E-66 | 3.1910063 | up |
| Glyma.16G151200.Wm82.a2.v1 | SNARE interactions in vesicular transport | ko04130 | 2 | 103 | 2.06E-102 | 2.8096829 | up |
| Glyma.01G179300.Wm82.a2.v1 | Endocytosis | ko04144 | 20 | 637 | 2.63E-25 | 1.2673699 | up |
| Glyma.11G062900.Wm82.a2.v1 | Endocytosis | ko04144 | 20 | 637 | 2.21E-25 | 1.2021647 | up |
