# Supplementary material for: Heterologous Expression of Arabidopsis AtARA6 in Soybean Enhances Salt Tolerance
Source: Front Genet. 2022 May 12;13:849357. doi: 10.3389/fgene.2022.849357 (PMC9134241; doi:10.3389/fgene.2022.849357)
Supplement: Supplementary file 10 [file Table3.docx]

**Supplementary Table 3_1 |** All GO enrichment of 1271 up-regulated DEGs

| **GO term** | **Ontology** | **Description** | **Genes** | **Total Genes** | **p-value** | **FDR** |
| --- | --- | --- | --- | --- | --- | --- |
| GO:0042221 | BP | response to chemical stimulus | 21 | 448 | 0.002 | 0.046 |
| GO:0050789 | BP | regulation of biological process | 91 | 2823 | 0.00051 | 0.012 |
| GO:0050794 | BP | regulation of cellular process | 91 | 2752 | 0.00022 | 0.0056 |
| GO:0016070 | BP | RNA metabolic process | 84 | 2485 | 0.00021 | 0.0054 |
| GO:0065007 | BP | biological regulation | 96 | 2931 | 0.0002 | 0.0054 |
| GO:0032774 | BP | RNA biosynthetic process | 80 | 2015 | 1.30E-06 | 4.00E-05 |
| GO:0006351 | BP | transcription, DNA-dependent | 80 | 2011 | 1.20E-06 | 4.00E-05 |
| GO:0060255 | BP | regulation of macromolecule metabolic process | 77 | 1886 | 7.80E-07 | 2.70E-05 |
| GO:0019222 | BP | regulation of metabolic process | 78 | 1898 | 5.20E-07 | 1.90E-05 |
| GO:0010468 | BP | regulation of gene expression | 77 | 1850 | 3.80E-07 | 1.60E-05 |
| GO:0080090 | BP | regulation of primary metabolic process | 77 | 1851 | 3.90E-07 | 1.60E-05 |
| GO:0051171 | BP | regulation of nitrogen compound metabolic process | 77 | 1825 | 2.30E-07 | 1.10E-05 |
| GO:0019219 | BP | regulation of Nuclear related metabolic process | 77 | 1811 | 1.70E-07 | 1.10E-05 |
| GO:0031323 | BP | regulation of cellular metabolic process | 78 | 1853 | 2.10E-07 | 1.10E-05 |
| GO:0031326 | BP | regulation of cellular biosynthetic process | 77 | 1816 | 1.90E-07 | 1.10E-05 |
| GO:0051252 | BP | regulation of RNA metabolic process | 77 | 1797 | 1.30E-07 | 1.10E-05 |
| GO:0010556 | BP | regulation of macromolecule biosynthetic process | 77 | 1816 | 1.90E-07 | 1.10E-05 |
| GO:0006355 | BP | regulation of transcription, DNA-dependent | 77 | 1796 | 1.20E-07 | 1.10E-05 |
| GO:0009889 | BP | regulation of biosynthetic process | 77 | 1816 | 1.90E-07 | 1.10E-05 |
| GO:0005623 | CC | cell | 133 | 3654 | 3.00E-08 | 1.70E-06 |
| GO:0044464 | CC | cell part | 133 | 3654 | 3.00E-08 | 1.70E-06 |
| GO:0003676 | MF | nucleic acid binding | 104 | 3180 | 0.00011 | 0.014 |
| GO:0043565 | MF | sequence-specific DNA binding | 28 | 516 | 3.90E-05 | 0.0067 |
| GO:0003700 | MF | transcription factor activity | 51 | 1002 | 1.80E-07 | 4.60E-05 |
| GO:0003677 | MF | DNA binding | 94 | 1992 | 3.30E-11 | 1.70E-08 |

**Supplementary Table 3_2 |** All GO enrichment of 2089 down-regulated DEGs

| **GO term** | **Ontology** | **Description** | **Genes** | **Total Genes** | **p-value** | **FDR** |
| --- | --- | --- | --- | --- | --- | --- |
| GO:0016052 | BP | carbohydrate catabolic process | 22 | 124 | 2.30E-06 | 0.00014 |
| GO:0006952 | BP | defense response | 20 | 96 | 7.50E-07 | 5.30E-05 |
| GO:0009308 | BP | amine metabolic process | 19 | 62 | 8.20E-09 | 7.70E-07 |
| GO:0044260 | BP | cellular macromolecule metabolic process | 386 | 6528 | 0.00072 | 0.019 |
| GO:0044267 | BP | cellular protein metabolic process | 247 | 3675 | 6.60E-06 | 0.00039 |
| GO:0055114 | BP | oxidation reduction | 175 | 2052 | 1.00E-10 | 1.30E-08 |
| GO:0009987 | BP | cellular process | 624 | 9924 | 9.00E-11 | 1.30E-08 |
| GO:0006066 | BP | alcohol metabolic process | 17 | 24 | 2.10E-12 | 3.40E-10 |
| GO:0006412 | BP | translation | 94 | 721 | 6.10E-15 | 1.10E-12 |
| GO:0006091 | BP | generation of precursor metabolites and energy | 51 | 208 | 8.70E-18 | 2.00E-15 |
| GO:0009765 | BP | photosynthesis, light harvesting | 26 | 36 | 2.10E-18 | 6.00E-16 |
| GO:0019684 | BP | photosynthesis, light reaction | 37 | 59 | 4.90E-24 | 1.80E-21 |
| GO:0015979 | BP | photosynthesis | 65 | 156 | 4.90E-33 | 5.50E-30 |
| GO:0043170 | BP | macromolecule metabolic process | 427 | 7301 | 0.00075 | 0.019 |
| GO:0044238 | BP | primary metabolic process | 529 | 9070 | 0.00013 | 0.0037 |
| GO:0019538 | BP | protein metabolic process | 281 | 4388 | 4.30E-05 | 0.0019 |
| GO:0044262 | BP | cellular carbohydrate metabolic process | 30 | 235 | 1.60E-05 | 0.00077 |
| GO:0006006 | BP | glucose metabolic process | 12 | 27 | 1.90E-07 | 1.60E-05 |
| GO:0016851 | MF | magnesium chelatase activity | 5 | 9 | 0.00037 | 0.014 |
| GO:0016705 | MF | oxidoreductase activity, acting on paired donors, with incorporation or reduction of molecular oxygen | 43 | 453 | 0.00019 | 0.0085 |
| GO:0016829 | MF | lyase activity | 31 | 285 | 0.00017 | 0.0082 |
| GO:0020037 | MF | heme binding | 55 | 624 | 0.00016 | 0.0078 |
| GO:0016835 | MF | carbon-oxygen lyase activity | 17 | 108 | 0.00012 | 0.0063 |
| GO:0046906 | MF | tetrapyrrole binding | 56 | 625 | 9.20E-05 | 0.0052 |
| GO:0051213 | MF | dioxygenase activity | 15 | 83 | 7.50E-05 | 0.0048 |
| GO:0003700 | MF | transcription factor activity | 81 | 1002 | 7.80E-05 | 0.0048 |
| GO:0016702 | MF | oxidoreductase activity, acting on single donors with incorporation of molecular oxygen, incorporation of two atoms of oxygen | 15 | 80 | 5.20E-05 | 0.0037 |
| GO:0016679 | MF | oxidoreductase activity, acting on diphenols and related substances as donors | 11 | 29 | 2.10E-06 | 0.00017 |
| GO:0004097 | MF | catechol oxidase activity | 9 | 16 | 1.50E-06 | 0.00013 |
| GO:0030414 | MF | peptidase inhibitor activity | 19 | 86 | 6.60E-07 | 6.30E-05 |
| GO:0004866 | MF | endopeptidase inhibitor activity | 19 | 86 | 6.60E-07 | 6.30E-05 |
| GO:0016682 | MF | oxidoreductase activity, acting on diphenols and related substances as donors, oxygen as acceptor | 11 | 22 | 2.40E-07 | 3.00E-05 |
| GO:0016701 | MF | oxidoreductase activity, acting on single donors with incorporation of molecular oxygen | 21 | 97 | 2.30E-07 | 3.00E-05 |
| GO:0019842 | MF | vitamin binding | 15 | 36 | 1.10E-08 | 1.90E-06 |
| GO:0005506 | MF | iron ion binding | 62 | 468 | 1.60E-10 | 3.40E-08 |
| GO:0016491 | MF | oxidoreductase activity | 195 | 2309 | 1.60E-11 | 4.50E-09 |
| GO:0005198 | MF | structural molecule activity | 91 | 576 | 4.10E-19 | 1.80E-16 |
| GO:0003735 | MF | structural constituent of ribosome | 87 | 532 | 3.80E-19 | 1.80E-16 |
