# Supplementary material for: Heterologous Expression of Arabidopsis AtARA6 in Soybean Enhances Salt Tolerance
Source: Front Genet. 2022 May 12;13:849357. doi: 10.3389/fgene.2022.849357 (PMC9134241; doi:10.3389/fgene.2022.849357)
Supplement: Supplementary file 11 [file Table4.docx]

| NaCl Concentration | WT | Line 1 | Line 2 | Line 3 |
| --- | --- | --- | --- | --- |
| 200mM | 2.543±0.085c | 4.923±0.208a | 4.703±0.150b | 4.823±0.211a |
| 100mM | 4.703±0.670d | 7.810±0.098b | 8.873±0.398a | 7.563±0.111c |
| 0mM | 10.320±0.437a | 10.580±0.168a | 10.490±0.243a | 10.460±0.083a |

**Supplementary Table 4_Root length of stress treatment during germination period**
