# Supplementary material for: Heterologous Expression of Arabidopsis AtARA6 in Soybean Enhances Salt Tolerance
Source: Front Genet. 2022 May 12;13:849357. doi: 10.3389/fgene.2022.849357 (PMC9134241; doi:10.3389/fgene.2022.849357)
Supplement: Supplementary file 12 [file Table6.docx]

**Supplementary Table 6_** **RNA-seq data of salt tolerance candidate genes identified from 935 DEGs**

| Gene | ID | Line2_Salt_Treatment_1 | Line2_Salt_Treatment_2 | SN9_Salt_Treatment_1 | SN9_Salt_Treatment_2 | FDR | log2FC | regulated |
| --- | --- | --- | --- | --- | --- | --- | --- | --- |
| Wrky6 | Glyma.08G142400.Wm82.a2.v1 | 558 | 552 | 50 | 107 | 7.18E-40 | -2.78935 | down |
| Wrky86 | Glyma.15G110300.Wm82.a2.v1 | 4131 | 3858 | 444 | 694 | 3.06E-254 | -2.80741 | down |
| MYC2 | Glyma.16G020500.Wm82.a2.v1 | 2959 | 2832 | 218 | 267 | 2.07E-248 | -3.54941 | down |
| Glyma_08G199300 | Glyma.16G020500.Wm82.a2.v1 | 5 | 11 | 3263 | 3195 | 1.8E-156 | -8.21161 | up |
| Glyma_07G126600 | Glyma.07G126600.Wm82.a2.v1 | 286 | 266 | 543 | 745 | 3.25E-15 | 1.21643 | up |
| Glyma_05G224500 | Glyma.05G224500.Wm82.a2.v1 | 140 | 168 | 4451 | 4090 | 0 | -4.75896 | up |
| Glyma_07G013900 | Glyma.07G013900.Wm82.a2.v1 | 98 | 206 | 1531 | 1558 | 4E-116 | -3.33536 | up |
